# Supplementary material for: Association between dietary inflammation index and herpes simplex virus I and II: A cross-sectional study
Source: PLoS One. 2025 Feb 12;20(2):e0316901. doi: 10.1371/journal.pone.0316901 (PMC11819573; doi:10.1371/journal.pone.0316901)
Supplement: Attachment — Submitted filename: Response to Reviewers.docx [file pone.0316901.s001.docx]

Response to Reviewers

Many thanks to the reviewers for their suggestions, and here are the revisions for each point.

To reviewer 1：

1. we found dietary inflammation index is not in MeSH, referring to other published articles on dietary inflammation index, we still put it in keywords, about which we can discuss further.

2. Thank you for your suggestion, it has been revised according to your suggestion.

3. Regarding the innovativeness of our article, we pointed out in the introduction of the manuscript that "No studies have yet examined the correlation between DII and herpes simplex virus infections among U.S. adults. S. adults. Therefore, exploring the correlation between DII and mono infections will provide new insights into the prevention and management of mono infections in the future. infections in the future."

4. the research design of our article is described in Statistical Analysis of Methods (line 248)

5. The study sample has been added (line 129)

6. grammatical errors in the discussion section have been corrected.

To reviewer 2：

1. the presentation of Abstract-Introduction has been revised

2. because DII-related data are only available for these five cycles in NHANES web.

3. the statement about education level has been revised (line 197).

4. specific diabetes testing indicators are mentioned in the official website of NHANES, do they need to be written in the body of the article? Diabetes related explanation has been modified.

5. Graphs and charts have been modified
