# Supplementary material for: Association between dietary inflammation index and herpes simplex virus I and II: A cross-sectional study
Source: PLoS One. 2025 Feb 12;20(2):e0316901. doi: 10.1371/journal.pone.0316901 (PMC11819573; doi:10.1371/journal.pone.0316901)
Supplement: Attachment — Submitted filename: Response to Reviewers(1).docx [file pone.0316901.s002.docx]

Response to Reviewers

Reviewer #2

1 Thank you very much for your advice, the stages of diabetes can be categorized into four, having diabetes, not having diabetes, impaired glucose tolerance (IGT) and impaired Fasting Glucose (IFG), by subdividing the diabetes stages to adjust for variables, as well as further stratified analyses at follow-up. In addition, there is more missing data for HbA1c, with a missing count of 8889 for 2007-2008, and the small sample size may not be conducive to analyzing the relationship between DII and herpes simplex virus.

2 Thank you for your suggestion, very professional, the data for our logistic regression analysis was weighted but missed count, now it has been added.

Reviewer #3

1. Thank you very much for your valuable suggestions, have added OR and CI values to the abstract section.

2. Thank you for your valuable suggestion, the introduction section has been modified.

3. Thank you for your suggestions, relevant references have been cited

4. Thank you for your valuable suggestions on this study. Regarding study gaps and novelty, this has been further clarified. In particular, the lack of studies on the relationship between DII and HSV infection among US adults has been emphasized. In addition, regarding the selection of the NHANES dataset, we have also provided more details in the manuscript.

5.We have added an explanation of the exclusion criteria in the Methods section, in addition to an explanation of all variables in the covariates section.

6. yes, we have adjusted for 45 components in the calculation of DII, of which 28 are food related.

7. The smoothed curve fit for herpes simplex virus and DII may be difficult to clearly see the correlation due to the small sample size, so we did not add it in the article.

8. Thank you for your suggestion, we have added specific p-values and other data.
